# Microsurgical resection of olfactory groove meningioma via the pterional transsylvian approach with preservation of the bilateral olfactory nerves

**DOI:** 10.3171/2025.10.FOCVID25181

**Published:** 2026-01-01

**Authors:** Semih Fidan, Moaz Ibrahim, Abuzer Güngör, Uğur Türe

**Affiliations:** 1 Department of Neurosurgery, Yeditepe University School of Medicine, İstanbul; and; 2 Faculty of Medicine, Department of Neurosurgery, Istinye University, İstanbul, Türkiye

**Keywords:** olfactory groove meningioma, anterior skull base surgery, pterional transsylvian approach

## Abstract

Olfactory groove meningiomas are slow-growing tumors arising from the dura of the cribriform plate and adjacent to the crista galli in the anterior cranial fossa. These tumors are frequently diagnosed late and may reach a very large size. Several surgical approaches have been described for olfactory groove meningiomas. The pterional transsylvian approach facilitates preservation of the olfactory nerves in most cases, allows for gross-total resection, and provides early access to vital structures. This video presents the successful treatment of a patient with an olfactory groove meningioma, with preservation of the bilateral olfactory nerves via the pterional transsylvian approach.

The video can be found here: https://stream.cadmore.media/r10.3171/2025.10.FOCVID25181

## Transcript

This video demonstrates the microsurgical resection of an olfactory groove meningioma via the pterional transsylvian approach^1^ while preserving the bilateral olfactory nerves.

### 0:33 Clinical Presentation.

A 56-year-old woman presented after a minor head injury, and imaging revealed an olfactory groove meningioma.

The neurological examination was unremarkable, with a normal bilateral sense of smell and taste.

### 0:50 Neuroimaging Findings.

MRI revealed a large, extra-axial, dural-based mass situated in the olfactory groove, extending to the planum sphenoidale, with homogeneous strong enhancement after contrast administration. The anteroposterior x-ray and CT scans demonstrated the presence of a large bilateral frontal sinus.

MR elastography allows evaluation of tissue stiffness and viscosity. In the displayed color scale, values closer to 0 indicate softer tissue, whereas values approaching 8 correspond to increasing stiffness.^2^ In this case, the lesion exhibited lower values, implying a soft consistency preoperatively.

### 1:39 Approach Selection—3D Visualization.

This segmented 3D holographic model illustrates the anatomical relationship between the lesion and the olfactory nerves.^3^ The olfactory nerves are shown in purple, and the tumor is shown in green. Several surgical techniques have been described for olfactory groove meningiomas. In the endoscopic endonasal approach, preservation of the olfactory nerves is not feasible, and there is a risk of cerebrospinal fluid leakage.^4–6^ The suprafrontal approach involves opening of the frontal sinus, with a risk of CSF leakage and may require transection of the superior sagittal sinus.^7^ In our case, we preferred the pterional transsylvian approach, which provides good orientation to the relationships between the meningioma and adjacent vital structures, while facilitating the preservation of the bilateral olfactory nerves and achieving gross-total resection.

### 2:43 Positioning.

The patient was positioned supine on the operating table, with the head secured in a 3-pin head holder and elevated approximately 15°.

### 2:54 Operative Techniques.

After the right-sided pterional craniotomy and dural opening, the frontal and temporal lobes as well as the sylvian fissure were visualized.

The superficial arachnoid membrane of the sylvian fissure was incised with the diamond knife, and the arachnoid was further opened with microscissors. Dissection was then continued toward the proximal portion of the sylvian fissure.

After opening the arachnoid layers of the carotid and chiasmatic cisterns, the right optic nerve, internal carotid artery, olfactory nerve, and the tumor were visualized.

Opening the proximal portion of the sylvian fissure and releasing CSF from the cisterns provides brain relaxation and creates a potential operative corridor without the need for rigid retraction. After dissecting the arachnoid bands and achieving brain relaxation, the tumor was found to displace the olfactory nerve laterally. The displaced and stretched olfactory nerve was then carefully dissected along the olfactory sulcus to prevent unintended avulsion. This maneuver allows access to frontobasally situated tumors while protecting the olfactory nerve from unnecessary retraction.^1^

### 4:16 Tumor Removal.

After dissection of the right-sided olfactory nerve, the tumor capsule was incised with microscissors, and part of the internal debulking was initiated. Following partial resection of the tumor, the dural attachment and vascular supply at the frontobasal dura were coagulated and dissected using bipolar cautery and suction. Dissection was performed along and over the right-sided olfactory nerve, extending toward the anterior frontobasal dura until the falx was encountered.

The junction of the falx with the crista galli was then exposed. The anterior part of the falx was carefully incised with a knife, providing access to the contralateral side. The left-sided olfactory nerve was identified, and a careful dissection of the tumor from the olfactory nerve was performed.

The tumor’s attachment to the frontobasal dura was sharply dissected using microscissors, and both olfactory nerves were subsequently observed to be free from the tumor. The attachments to the crista galli and anterior falx were divided, providing complete control of the contralateral extension of the tumor. Then the tumor portion extending to the crista galli was removed. Finally, the tumor was dissected from the left gyrus rectus.

After dissection of the tumor from the frontobasal dura, interruption of its main vascular supply, and preservation of both olfactory nerves, removal of the softened lesion was initiated in a piecemeal fashion. First, the portion extending into the interhemispheric area was excised, followed by careful dissection of the adherent parts from the brain parenchyma. Use of rigid retractors for a long duration may cause frontal lobe contusion and edema. Therefore, brief retraction was applied to remove the tumor portion extending into the interhemispheric area. Finally, the remaining tumor tissue was carefully removed.

After completion of the gross-total resection, the chiasmatic cistern was opened, and after the blood was cleared, the optic chiasm and bilateral optic nerves were exposed. The frontobasal dura, the site of tumor attachment, was coagulated with monopolar cautery and then resected. The underlying frontobasal bone at the tumor attachment site was carefully drilled. Following hemostasis, both olfactory nerves were observed to be preserved.

### 8:27 Closure.

Finally, the dura was closed in a watertight fashion

### 8:33 Outcome.

Intraoperative MRI confirmed gross-total resection. A follow-up MRI obtained at 3 months showed no evidence of residual tumor. On neurological examination, the bilateral sense of smell remained normal, as confirmed by testing each nostril separately while occluding the other, using a nonirritating substance, with the eyes closed. Histopathological analysis confirmed the meningothelial meningioma of WHO grade 1.

### 9:08 Conclusion.

Olfactory groove meningiomas are slow-growing tumors that arise from the cribriform plate adjacent to the crista galli, and are often diagnosed late. These tumors often produce severe compression and displacement of the olfactory nerves. In tumors extending from the frontobasal part of the interhemispheric fissure, the pterional approach facilitates the mobilization and preservation of the olfactory nerve from the olfactory sulcus. The pterional transsylvian approach allows clear visualization of the meningioma and adjacent vital structures near the sella, without severe frontal lobe retraction.

Thank you.

## Acknowledgments

We thank Aylin Akçaoğlu for artistic illustrations.

## Disclosures

The authors report no conflict of interest concerning the materials or methods used in this study or the findings specified in this publication.

## Author Contributions

Primary surgeon: Türe. Assistant surgeon: Fidan, Ibrahim. Editing and drafting the video and abstract: all authors. Critically revising the work: Türe, Güngör. Reviewed submitted version of the work: Türe, Güngör. Approved the final version of the work on behalf of all authors: Türe. Supervision: Türe.

## Supplemental Information

### Patient Informed Consent

The necessary patient informed consent was obtained in this study.
